# Activation of the DNA-repair mechanism through NBS1 and MRE11 diffusion

**DOI:** 10.1371/journal.pcbi.1006362

**Published:** 2018-07-27

**Authors:** Ida Friis, Ilia A. Solov’yov

**Affiliations:** 1 Department of Physics, Chemistry and Pharmacy, University of Southern Denmark, Campusvej 55, 5230 Odense M, Denmark; 2 On leave from the Ioffe Institute, Politechnicheskaya Str. 26, 94021, St. Petersburg, Russia; Rutgers University, UNITED STATES

## Abstract

The non-homologous end joining of a DNA double strand break is initiated by the MRE11-NBS1-RAD50 complex whose subunits are the first three proteins to arrive to the breakage site thereby making the recruitment time of MRE11, NBS1 and RAD50 essential for cell survival. In the present investigation, the nature of MRE11 and NBS1 transportation from the cytoplasm to the nucleus, hosting the damaged DNA strand, is hypothesized to be a passive diffusive process. The feasibility of such a mechanism is addressed through theoretical and computational approaches which permit establishing the characteristic recruitment time of MRE11 and NBS1 by the nucleus. A computational model of a cell is constructed from a set of biological parameters and the kinetic Monte Carlo algorithm is used to simulate the diffusing MRE11 and NBS1 particles as a random walk process. To accurately describe the experimented data, it is discovered that MRE11 and NBS1 should start diffusion from significantly different starting positions which suggests that diffusion might not be the only transport mechanism of repair protein recruitment to the DNA break.

## Author summary

The DNA repair mechanism is crucial for a cell to avoid apoptosis, and is a complicated process involving many different repair proteins. The mean of transportation of these repair proteins is largely unknown as their transportation mechanisms need clarification. We have focused on the transportation of some of the first proteins to arrive to the damaged DNA from the cytoplasm, namely the MRE11 and NBS1. Our hypothesis of diffusion as the transportation mechanism of MRE11 and NBS1 is tested against a theoretical model as well as simulation data and experiments, revealing a possibility for diffusion to be the method of recruiting MRE11 and NBS1 to the DNA break.

## Introduction

Once a DNA strand inside a cell nucleus breaks, the ruptured strands are sought mended by a biological multi-step process [1–4]. This breakage and repair can happen in various different ways. The double strand breaks are for example repaired by non-homologous end-joining (NHEJ), where broken ends are directly joined without the need of a template DNA [5–7]. NHEJ has the advantage that it occurs in every cell stage [8], and is therefore a stable repair mechanism throughout the cell cycle.

DNA breakage can, for example, be induced by ionizing radiation which is used in radiotherapy, a certain kind of cancer treatment [9, 10]. The DNA-repair is a crucial mechanism in the cell, as failing to mend the breakage will ultimately lead to apoptosis, killing the cell [11], or alternatively end in misrepair and mutations that potentially can induce cancer tumors. Knowledge about how long the repair process takes can be important for planning the course of a radiotherapy treatment, aiding in constructing the so-called survival curves, a tool that predicts the survival of the cell as a function of radiation dose [12–14].

The multi-step mechanism of NHEJ DNA-repair could be initiated by the arrival of the proteins MRE11, RAD50 and NBS1, some of the first proteins to arrive to the broken DNA-ends, where they form the so-called MRN-complex [3, 15]. The exact protein to initiate the DNA repair mechanism could in principle be the MRN-complex, but other models have been proposed as well. It is speculated that the protein PARP1 initiates the repair process by modifying the chomatin structure around the breakage site [16], or that the KU70/KU80 heterodimer immediately is recruited to stabilize the broken DNA ends [17]. It is, however, quite established that the recruitment of the MRN-complex is crucial for the signaling process of DNA damage response. MRE11 especially interacts with and activates ATM (ataxia-telangiectasia mutated) and ATR (ATM- and RAD3-related) [18], the proteins responsible for phospholation of the proteins participating in the DNA damage repair, and thus regulate the repair mechanism [19]. While the details of ATM’s role in activating other proteins and enzymes is still not fully understood, it is generally agreed that without this protein kinase cascade, the repair process would not take place.

**Fig 1:**
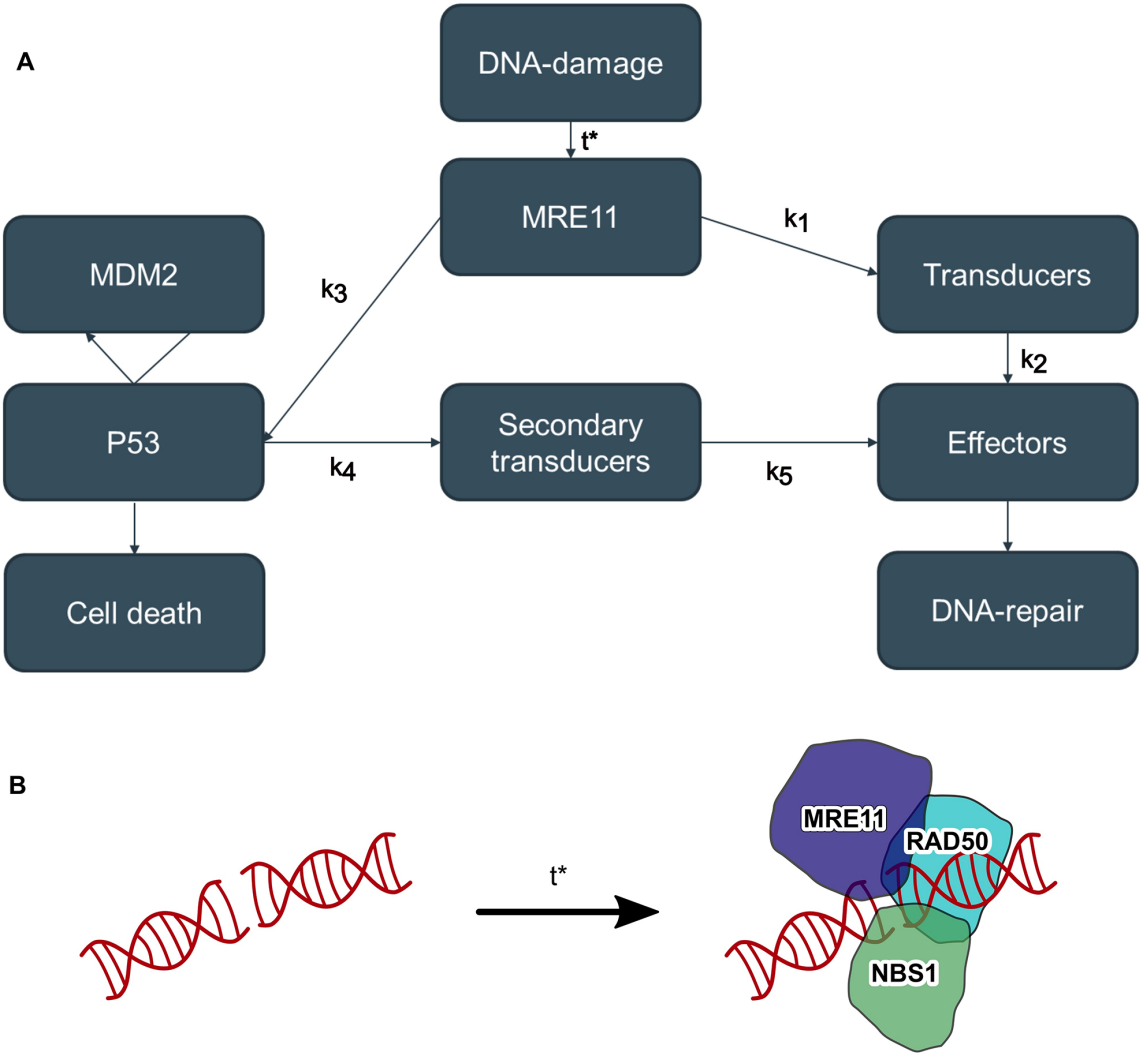
Flow diagram of the DNA-repair process. **A:** The average time of MRE11, NBS1 or RAD50 getting recruited to the DNA-damage site is denoted t* and consists of the characteristic time of all three of MRE11, RAD50 and NBS1. The different activation rate constants are labelled *k*_1−5_. These processes include phosphorylation which starts protein kinase cascades. **B:** The broken DNA strand recruits proteins MRE11, NBS1, and RAD50 after a characteristic time, *t*_*N*_, *t*_*M*_ and *t*_*R*_ respectively. The combined recruitment time is denoted t*.

The DNA is located inside the nucleus whereas proteins, including MRE11, RAD50 and NBS1, are made outside the nucleus by the ribosomes [20]. The arrival of MRE11, RAD50 and NBS1 to the DNA-nick results in the formation of the MRN-complex which starts a protein kinase cascade, where a chain of transducer proteins are phosphorylated to turn to their active state, ending up activating the effector proteins, which are the proteins directly responsible for repairing the DNA. [21] On the other hand, the recruitment of MRE11, RAD50 and NBS1 to a DNA-break will activate the protein P53 as well; if the concentration of P53 gets too high, it will trigger cellular apoptosis [22]. To start the DNA-repair, the MRE11, RAD50 and NBS1 need to be transported to the nucleus, a process of which the time duration is the first factor of the time scale of the entire repair process. The individual recruitment times of MRE11, RAD50 and NBS1 proteins are denoted *t*_*N*_, *t*_*M*_ and *t*_*R*_, respectively, and the combined characteristic recruitment time of all three of them is t*. This combined characteristic time, t*, of the recruitment of MRE11, RAD50 and NBS1 to a DNA-nick along with the rates of activation *k*_1_ − *k*_5_ govern the time duration of a DNA break repair. The overall process is illustrated in a specific scheme in Fig 1. It is, however, remarkable that the mechanisms of MRE11, RAD50 and NBS1 transportation into the nucleus is currently unclear, and could for example be diffusive in nature or involve active transporters [23, 24]. The question whether the time of MRE11, RAD50 and NBS1 cumulative recruitment could be explained by simple diffusion is the main focus of the present investigation. In the crowded cellular environment the simple diffusion process has been dismissed as a possible model to describe attachment of proteins. Numerically, subdiffusion [25] has been used as a computational model, while biochemically active transport processes are considered to be more realistic [24]. In the latter case the desired protein is transported into the nucleus while being assisted by e.g. GTPases [26].

For completeness it should be mentioned that the success and time scale of DNA repair is influenced by several factors other than the rapid recruitment of specific proteins. Such other factors cold be the higher order chromatin structure domains around the DNA nick, where the density of the chromatin changes the radiosensitivity of the irradiated cell, [27], as well as the complexity of the DNA breaks can alter the repair time [28].

The present study will address if ordinary diffusion theory is capable of explaining MRE11 and NBS1 transportation by comparing experimental, theoretical and simulation data. RAD50 transportation is omitted from the investigation due to a lack of experimental data that would be comparable to the one used for characterizing MRE11 and NBS1 dynamics. [16]. The simulation of MRE11 and NBS1 diffusion is carried out by employing kinetic Monte Carlo simulations and the theoretical data is obtained from calculating the so-called MRE11 and NBS1 first passage time [29–31], which permits evaluating the time it takes for a Brownian particle to reach a defined target.

## Materials and methods

### Cellular model

**Fig 2:**
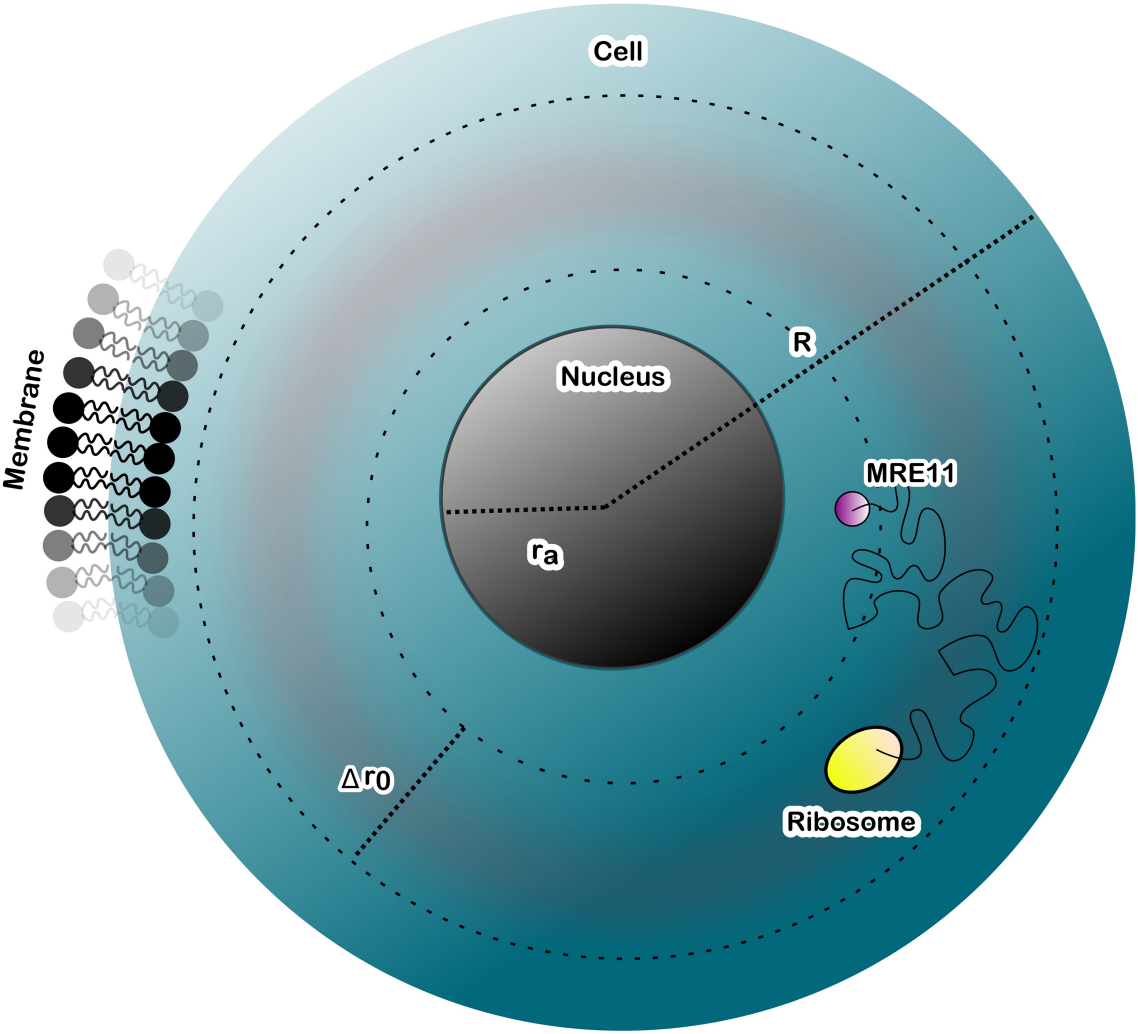
Sketch of the studied cell model. The outer shell has a radius *R* = 25 *μ*m, and the outer membrane is modelled through reflective boundary conditions. MRE11 and NBS1 is modelled as a Brownian particle and is initially made by the ribosome which is placed at a radial distance within Δ*r*_0_ which is within the range 11-20 *μ*m away from the center of the nucleus. The nucleus has a radius of *r*_*a*_ = 5 *μ*m, and is modelled as an absorbing sphere due to the nuclear pore complexes on its surface.

The biological environment of the damaged DNA is important to be taken into consideration when studying MRE11 and NBS1 diffusion. In this study a simple model of a cell used to describe the essential biophysics of the MRE11 and NBS1 diffusion process is considered. The biological system is constructed following the known conditions of NBS1 and MRE11 diffusing in eukaryotic cells, modeled after a human bone marrow cell [32]. Since the cellular membrane is largely impermeable in the suggested model, all of the MRE11 and NBS1 proteins are contained inside the cell, and should they hit the membrane, they will bounce of it again. However, if the proteins hit the nucleus, passage would be permitted by the nuclear pore complexes, which are considered to be spread uniformly on the surface of the nucleus [33]. MRE11 and NBS1 proteins are produced in the ribosomes located outside the nucleus. To construct the simple theoretical model of the cell, consider a spherical topology, where the outer membrane is placed at a distance *R*, the ribosome at a distance *r*_0_ and the inner membrane is placed at a distance *r*_*a*_ from the cell center. Of course, the free ribosomes in the cytoplasm are not located in one specific spot, but can be found within a range from the cell center. A schematic cell model is shown in Fig 2, while the biologically relevant parameters are compiled in Table 1.

**Table 1:** Summary of biological parameters of MRE11 and NBS1 transportation. The cell is modelled after the human bone marrow cell (osteoblast) [32], and the diffusion coefficients, *D*_NBS1_, for NBS1 of 85 kDa and *D*_MRE11_ for MRE11 of 80 kDa are found in the literature. [34, 35].

*R*(*μ*m)	*r*_*a*_ (*μ*m)	*r*_0_ (*μ*m)	*D*_NBS1_ (*μ*m^2^/s)	*D*_MRE11_ (*μ*m^2^/s)
25	5.0	11-20	2.5	2.0

#### Theoretical model

The theoretical formulation of MRE11 and NBS1 diffusion can be used to describe the number of MRE11 and NBS1 proteins that become recruited by the nucleus as a function of time. In this case, the so-called first passage time describes the time it takes for a freely diffusing particle to reach a target point [29–31, 36, 37]. The first passage time for a particle representing either the MRE11 or the NBS1 to hit the nucleus is related to the characteristic time t*, introduced in Fig 1. The latter relies on the individual recruitment times of MRE11 and NBS1, denoted as *t*_M_ and *t*_N_, respectively. As diffusion is a stochastic process, the characteristic time is not an exact value, but its statistics can be determined as a distribution, which can be found as the probability flux into the desired target, $P(t_{\text{M}})$ or $P(t_{\text{N}})$, depending on the protein. Once the probabilities of the characteristic MRE11 and NBS1 recruitment times have been obtained, this result is integrated to find the accumulated number of hits, *N*, which can be compared with experimental data of MRE11 and NBS1 recruitment. [16]

The theoretical distribution of characteristic times, $P(t_{i})$, is calculated by describing the diffusion as a random walk process and solving for the first passage time of a spherically symmetric system constructed of an outer sphere with reflecting boundary conditions with a radius of *R* = 25 *μ*m and a inner sphere with absorbing boundary conditions and a radius of *r*_*a*_ = 5 *μ*m, as seen in Fig 2 and Table 1, to reflect the properties of a cell. The probability density function for MRE11 and NBS1 proteins to have traveled the distance *r* during a time *t* is denoted *P*(*r*, *t*) and is governed by the diffusion equation for the spherically symmetric system, which reads as:

$$\begin{array}{c} \frac{\partial }{\partial t}P\left(r,t_{i}\right)=\frac{D_{i}}{r^{2}}\frac{\partial }{\partial r}r^{2}\frac{\partial }{\partial r}P\left(r,t_{i}\right), \end{array}$$ (1)

where *D*_*i*_, (*i* = MRE11, NBS1) is the diffusion constant for either MRE11 or NBS1, respectively. The subscript *i* is omitted in the following for the sake of brevity. To solve this differential equation, it is necessary to transform the time-variable, *t*, into the Laplace variable, λ, and impose the boundary conditions for one reflective outer boundary and one absorbing inner boundary, as:

$$\frac{\partial P\left(r,t\right)}{\partial r}|{}_{R}=0\;\text{and}\;P\left(r_{a},t\right)=0.$$ (2)

Introducing

$$\begin{array}{c} x=r\sqrt{\frac{\lambda }{D}},\;x_{0}=r_{0}\sqrt{\frac{\lambda }{D}},\;x_{a}=r_{a}\sqrt{\frac{\lambda }{D}},\;x_{R}=R\sqrt{\frac{\lambda }{D}}, \end{array}$$ (3)

it is possible to construct the Green’s function for the diffusion equation that will yield a non-zero solution as [29]:

$$\begin{array}{c} G\left(x,\lambda \right)=\frac{\sqrt{\lambda }}{4\pi D^{3/2}\sqrt{xx_{0}}}\frac{C_{-\frac{1}{2}}(x_{0},x_{a})D_{-\frac{1}{2}}(x,x_{R})}{D_{-\frac{1}{2}}(x_{a},x_{R})}. \end{array}$$ (4)

The Green’s function in Eq (4) permits calculating the probability flux of MRE11 and NBS1 into the nucleus as the surface integral over the inner sphere with the radius *r*_*a*_:

$$P\left(\lambda \right)=\underset{S}{\int }D\frac{\partial G}{\partial r}dA|{}_{r=r_{a}}.$$ (5)

The notations $D_{-\frac{1}{2}}$ and $C_{-\frac{1}{2}}$ in Eq (4) are the special functions defined as [29]:

$$D_{-\frac{1}{2}}(x_{a},x_{R})=I_{-\frac{1}{2}}\left(x_{a}\right)K_{-\frac{3}{2}}\left(x_{R}\right)+K_{-\frac{1}{2}}\left(x_{a}\right)I_{-\frac{3}{2}}\left(x_{R}\right)$$ (6)

$$D_{-\frac{1}{2}}(x,x_{R})=I_{-\frac{1}{2}}\left(x\right)K_{-\frac{3}{2}}\left(x_{R}\right)+K_{-\frac{1}{2}}\left(x\right)I_{-\frac{3}{2}}\left(x_{R}\right)$$ (7)

$$C_{-\frac{1}{2}}(x_{0},x_{a})=I_{-\frac{1}{2}}\left(x_{0}\right)K_{-\frac{1}{2}}\left(x_{a}\right)-K_{-\frac{1}{2}}\left(x_{0}\right)I_{-\frac{1}{2}}\left(x_{a}\right),$$ (8)

where $I_{-\frac{1}{2}}$ and $K_{-\frac{1}{2}}$ are the Bessel functions of the first and second order, respectively. The differential in Eq (5) can be found in terms of the radial coordinates as:

$$\begin{array}{ccc} \frac{\partial G}{\partial r} & = & \frac{1}{4D\pi r^{2}r_{0}}\sinh((R-r_{0})\sqrt{\frac{\lambda }{D}})\times \\ & & (\frac{\left(r_{a}-r)\sqrt{\lambda D}\cosh(\left(r-r_{a}\right)\sqrt{\frac{\lambda }{D}})+(D-rr_{a}\lambda )\sinh(\left(r-r_{a}\right)\sqrt{\frac{\lambda }{D}}\right)}{r_{a}\lambda \cosh(\left(R-r_{a}\right)\sqrt{\frac{\lambda }{D}})+\sqrt{\lambda D}\sinh(\left(R-r_{a}\right)\sqrt{\frac{\lambda }{D}})}), \end{array}$$ (9)

which, after evaluating the integral in Eq (5), permits writing the probability flux into the nucleus in Laplace space as:

$$\begin{array}{c} P\left(\lambda \right)=\frac{r_{a}(R\sqrt{\frac{\lambda }{D}}\cosh(\left(R-r_{0}\right)\sqrt{\frac{\lambda }{D}})-\sinh(\left(R-r_{0}\right)\sqrt{\frac{\lambda }{D}}))}{r_{0}(R\sqrt{\frac{\lambda }{D}}\cosh(\left(R-r_{a}\right)\sqrt{\frac{\lambda }{D}})-\sinh(\left(R-r_{a}\right)\sqrt{\frac{\lambda }{D}}))}. \end{array}$$ (10)

The probability density of MRE11 and NBS1 diffusion time in real space can thus be calculated by taking the inverse Laplace transformation of Eq (10), however the analytical expression is hardly possible.

According to the finite value theorem [38], the asymptotic in Laplace and real spaces can be related for the case of real time approaching infinity, corresponding to the Laplace variable λ → 0. In this case Eq (10) can be rewritten as:

$$\begin{array}{ll} P\left(\lambda \right)= & (\frac{r_{0}R\sqrt{\frac{\lambda }{D}}\text{cosh}\left(\left(R-r_{a}\right)\sqrt{\frac{\lambda }{D}}\right)}{r_{a}\left(R\sqrt{\frac{\lambda }{D}}\text{cosh}\left(\left(R-r_{0}\right)\sqrt{\frac{\lambda }{D}}\right)-\text{sinh}\left(\left(R-r_{0}\right)\sqrt{\frac{\lambda }{D}}\right)\right)}-\\ & {\frac{r_{0}\text{sinh}\left(\left(R-r_{a}\right)\sqrt{\frac{\lambda }{D}}\right)}{r_{a}\left(R\sqrt{\frac{\lambda }{D}}\text{cosh}\left(\left(R-r_{0}\right)\sqrt{\frac{\lambda }{D}}\right)-\text{sinh}\left(\left(R-r_{0}\right)\sqrt{\frac{\lambda }{D}}\right)\right)})}^{-1}, \end{array}$$ (11)

which allows constructing the asymptotically correct Taylor expansion of each of the two terms in the nominator for the small values of λ yielding:

$$\begin{array}{c} P\left(\lambda \right)=\frac{6Dr_{0}r_{a}}{6Dr_{0}r_{a}-\lambda (4R^{4}+12R^{2}r_{0}r_{a}-2R^{3}\left(5r_{0}+r_{a}\right)-r_{0}r_{a}\left(r_{0}^{2}-r_{a}^{2}\right)+2R\left(r_{0}^{3}-3r_{0}r_{a}^{2}\right))}. \end{array}$$ (12)

The inverse Laplace transformation of P(λ) in Eq (12) delivers the distribution for the slow MRE11 or NBS1 diffusion, which is expected to describe the tail of the $P(t_{i})$ distribution (where *i* = MRE11, NBS1), depending on the diffusion coefficient *D*_*i*_:

$$\begin{array}{cc} P_{SD}\left(t_{i}\right) & =\frac{6D_{i}r_{0}r_{a}\exp(-\frac{6D_{i}r_{0}r_{a}}{-4R^{4}+10R^{3}r_{0}-2Rr_{0}^{3}+2R^{3}r_{a}-12R^{2}r_{0}r_{a}+r_{0}^{3}r_{a}+6Rr_{0}r_{a}^{2}-r_{0}r_{a}^{3}}t_{i})}{-4R^{4}+10R^{3}r_{0}-2Rr_{0}^{3}+2R^{3}r_{a}-12R^{2}r_{0}r_{a}+r_{0}^{3}r_{a}+6Rr_{0}r_{a}^{2}-r_{0}r_{a}^{3}}= \end{array}$$ (13)

$$\begin{array}{c} =-\frac{D_{i}r_{0}}{1.5r_{0}^{3}-4079r_{0}+46875}\exp(\frac{D_{i}r_{0}}{1.5r_{0}^{3}-4079r_{0}+46875}t_{i}). \end{array}$$ (14)

The numerical expression in Eq (14) is obtained by utilizing the biological values from Table 1 and assuming different values of the protein starting position, defined by *r*_0_. The expression reveals that the tail of the distribution $P(t_{i})$ of recruitment times of MRE11 or NBS1 is decaying exponentially. Here *D*_*i*_ is measured in *μ*m^2^/s and *t*_*i*_ is measured in seconds.

In the case of the Laplace parameter λ → ∞, one can invoke the initial value theorem [38], which states that this case is equivalent to *t* → 0 in real time, and the hyperbolic functions in Eq (10) can be rewritten asymptotically as:

$$\begin{array}{c} \lim_{t\to \infty }(\cosh(t))∼\frac{e^{t}}{2},\;\lim_{t\to \infty }(\sinh(t))∼\frac{e^{t}}{2}. \end{array}$$ (15)

By inserting these asymptotic values into Eq (10), one obtains:

$$\begin{array}{c} P\left(\lambda \right)=\frac{r_{a}}{r_{0}}\exp(\left(r_{a}-r_{0}\right)\sqrt{\frac{\lambda }{D}}). \end{array}$$ (16)

Note that the expression in Eq (16) does not contain the outer boundary, *R*, as the fast diffusion does not involve an interaction with the cellular membrane. The inverse Laplace transformation of Eq (16) yields:

$$P_{FD}\left(t_{i}\right)=\sqrt{\frac{4}{D_{i}}}\frac{\left(r_{0}-r_{a}\right)r_{a}}{\sqrt{\pi }r_{0}t_{i}^{3/2}}\exp(-\frac{{\left(r_{0}-r_{a}\right)}^{2}}{4D_{i}t_{i}})=$$ (17)

$$=0.28\frac{1}{\sqrt{D_{i}t_{i}^{3}}}(r_{0}-5)\exp(-\frac{{\left(r_{0}-5\right)}^{2}}{4D_{i}t_{i}}),$$ (18)

which shows that the fast MRE11 or NBS1 recruitment follows a Lévy distribution.

Another useful characteristic to describe MRE11 and NBS1 recruitment is the accumulated number of proteins reaching the nucleus as a function of time, which can be obtained by integrating Eqs (18) and (14) to obtain the functions *N*_*FD*_ and *N*_*SD*_, respectively. Both quantities can readily be compared to the recruitment curves found experimentally [16]. Integration of Eq (14) yields:

$$\begin{array}{c} N_{SD}\left(t_{i}\right)=\int P_{SD}dt=1-\exp(\frac{D_{i}r_{0}}{1.5r_{0}^{3}-4079r_{0}+46875}t_{i}), \end{array}$$ (19)

where *t*_*i*_ is measured in seconds and the value 1 is an integration constant found by enforcing the boundary condition for the longer times:

$$\begin{array}{c} \lim_{t_{i}\to \infty }N_{SD}\left(t_{i}\right)=1. \end{array}$$ (20)

Likewise the fast MRE11 or NBS1 recruitment, Eq (18) can be integrated to obtain:

$$\begin{array}{c} N_{FD}\left(t_{i}\right)=\int P_{FD}dt=\frac{20}{r_{0}}(\text{erf}(\frac{5-r_{0}}{2\sqrt{D_{i}t_{i}}})+1), \end{array}$$ (21)

where the integration constant 1 arises from the boundary condition:

$$\begin{array}{c} \lim_{t_{i}\to 0}N_{FD}\left(t_{i}\right)=0. \end{array}$$ (22)

The characteristic limiting upper time of the fast diffusion can be obtained from the well known equation for a three dimensional diffusion: [39]

$$\Delta t=\frac{\langle L^{2}\rangle }{6D},$$ (23)

where 〈*L*^2^〉 is the average square displacement of MRE11 or NBS1 during the time Δ*t* and *D* is the diffusion coefficient for the protein diffusion in the cellular environment, given in Table 1. By choosing the starting position of both proteins to be *r*_0_ = 20 *μ*m $≃\sqrt{\langle L^{2}\rangle }$, the fast diffusion regime is expected to be applicable for *t* ≲ 27 s in the case of NBS1 recruitment and *t* ≲ 33 s in the case of MRE11 recruitment.

#### Simulation details

The stochastic motion of MRE11 and NBS1 protein is alternatively simulated by employing the kinetic Monte Carlo approach [40, 41] using MBN Explorer [42], and the first hitting time is measured from the produced trajectories. The kinetic Monte Carlo algorithm works by defining a 3D grid for coarse grained MRE11 and NBS1 particles to move on. The diffusing particles are randomly translated from one grid unit to another with equal translation probability in each direction. The time interval, Δ*t*, it takes for one protein (either MRE11 or NBS1) to diffuse from one grid unit into another can be calculated from Eq (23), where, in this case, 〈*L*^2^〉 is the average square displacement of MRE11 or NBS1 during one simulation step.

For the numerical simulations of MRE11 and NBS1 diffusion, a model of a cell is approximated as a parallelepiped with the dimensions 50×50 ×45 *μ*m^3^ having reflective boundary conditions. The cell is discretized in 1300×1300×1200 units of 38.5×38.5×37.5 nm^3^ each. Herein a spherical nucleus is constructed with a radius of *r*_*a*_ and MRE11 and NBS1 emerging from the ribosome are simulated independently until the protein hits the nucleus. This process is repeated 4200 times each simulation being 1800 s long, allowing to measure MRE11 and NBS1 slow diffusion. In the limit of fast diffusion for MRE11 or NBS1, the size of the grid units are decreased to increase the precision of the simulations, such that each grid unit is assumed to have the size of 20×20×20 nm^3^ size and yet another 2200 simulations were carried out, each for a time duration of 100 s. The large number of simulation replicas is needed to create sufficient statistics in describing the first passage times of MRE11 and NBS1 to the nucleus in the two considered diffusion regimes. The placement of a single particle at every simulation event implies that no concentration gradient is created. Therefore, every single one of the 2200 simulations rely exclusively on the random Brownian motion of the protein induced by the small solvent molecules of the cytoplasm in which the protein is submerged.

Since two different simulations grids are used, two characteristic NBS1 diffusion time intervals emerge, namely Δ*t*_*FD*_ = 9.88 ⋅ 10^−5^ s and Δ*t*_*SD*_ = 2.66 ⋅ 10^−4^ s for NBS1 and Δ*t*_*FD*_ = 3.33 ⋅ 10^−5^ s and Δ*t*_*SD*_ = 1.23 ⋅ 10^−4^ s for MRE11, representing the characteristic time intervals for one simulation step in both simulations. The simulations are repeated for both the fast and slow diffusion process for several starting positions within the range for free ribosomes as outlined in Table 1.

## Results

The results of NBS1 diffusion and first passage times from the cytoplasm to the nucleus, obtained from kinetic Monte Carlo simulations are compared to the theoretical predictions and numerical results from both MRE11 and NBS1 are compared to results from experiments.

**Fig 3:**
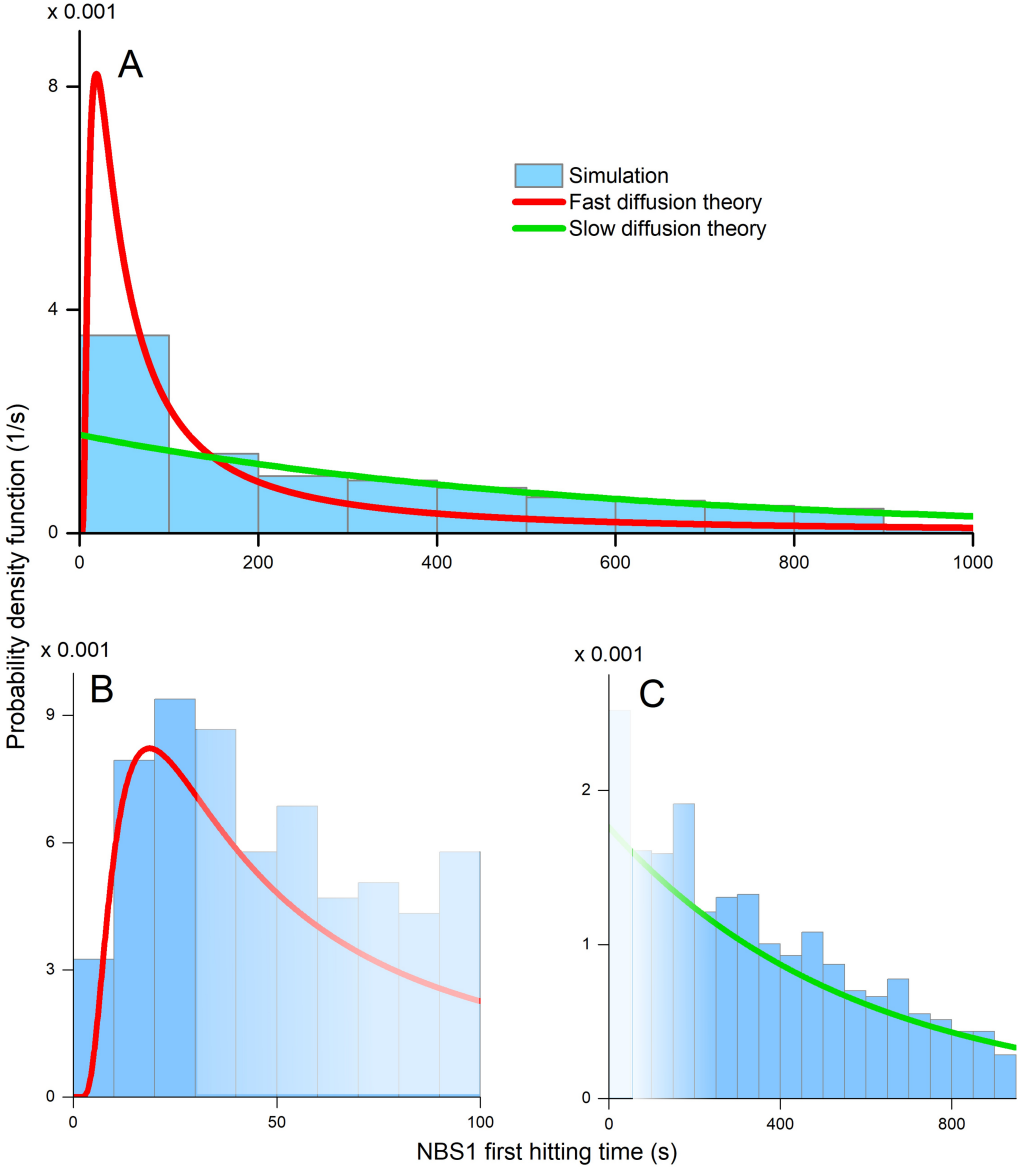
Distribution for the first passage times of NBS1 protein. **A**: The blue histogram shows the first passage times of NBS1 obtained by simulations. The red line shows the approximation for fast diffusion, and the green line shows the theoretical approximation for slow diffusion theory. None of the distributions fits the entire spectrum of the first hitting times for NBS1. **B:** The blue histogram shows the simulated first passage time of fast NBS1 recruitment by the nucleus and the red line shows the theoretical probability distribution for the first hitting time, which follows from Eq (18). The faded area marks the time interval outside the validity of the theoretical approximation. **C:** The blue histogram shows the slow first passage time of NBS1 being recruited to the nucleus and the green line shows the theoretical probability distribution for the slow first hitting time, predicted using Eq (14). The faded area marks the time interval outside the validity of the theoretical approximation.

Fig 3 shows the histograms of the first passage times, obtained from kinetic Monte Carlo simulations, and provides a comparison to the probability density functions (PDFs), described by Eqs (14) and (18) for NBS1. It is evident that replicating the NBS1 diffusion process inside a cell multiple times results in satisfactory statistics which leads to a reasonable agreement between theory and simulations, and furthermore that the simulations diverge rapidly from the theory predictions as the time progresses beyond regimes validated by the theory of diffusion.

The distribution of the fast NBS1 recruitment time shown in Fig 3B, features a distinct peak at 22 s as predicted by the Levy distribution in Eq (18), while Fig 3C shows that the first hitting times of the nucleus by the NBS1 greater than 300 s obtained from simulations, are correctly described by Eq (14), which is an exponential distribution that does not apply to the behaviour at small values of *t*_*N*_. Note that neither Eq (18), nor Eq (14), are applicable for describing the intermediate recruitment times, see Fig 3A, as the general solution of Eq (1) becomes problematic. To describe the non-asymptotic times regions experimental measurements or simulations are needed.

**Fig 4:**
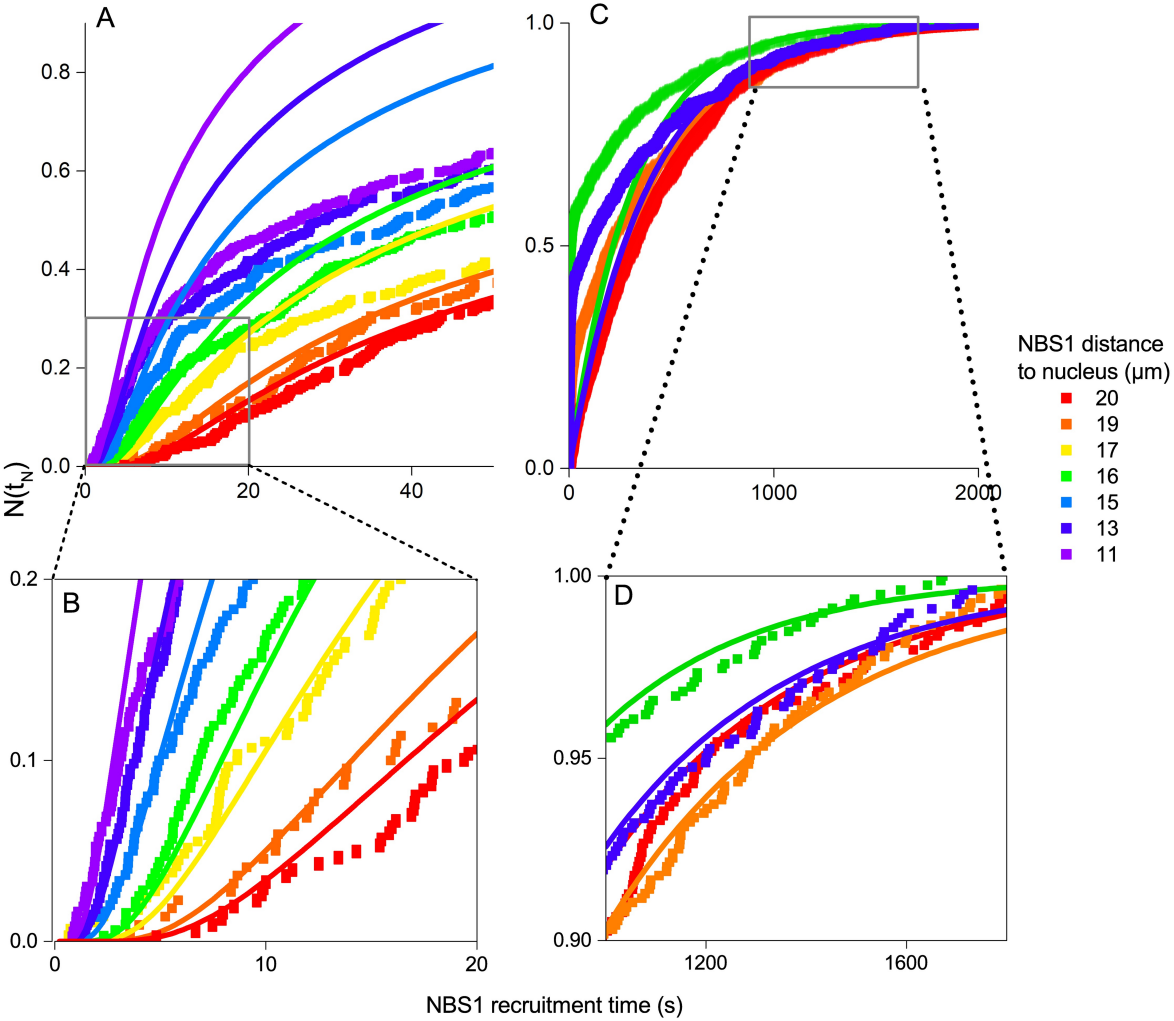
Accumulated fraction of NBS1 in the nucleus. **A**: The lines represent the faction of accumulated NBS1 proteins in the nucleus, *N*(*t*_*N*_) defined by Eq (21). The squares represent the corresponding numerical results for the different starting points. In the short time limit, the two methods agree, which is further highlighted in the insert **B**. **C:** The lines represent the faction of accumulated NBS1 proteins in the nucleus for the slow diffusion, *N*(*t*_*N*_) defined by Eq (19). The squares represent the corresponding numerical results for the different starting points. In the long time limit, the two methods agree, as evidenced in the insert **D**.

**Fig 5:**
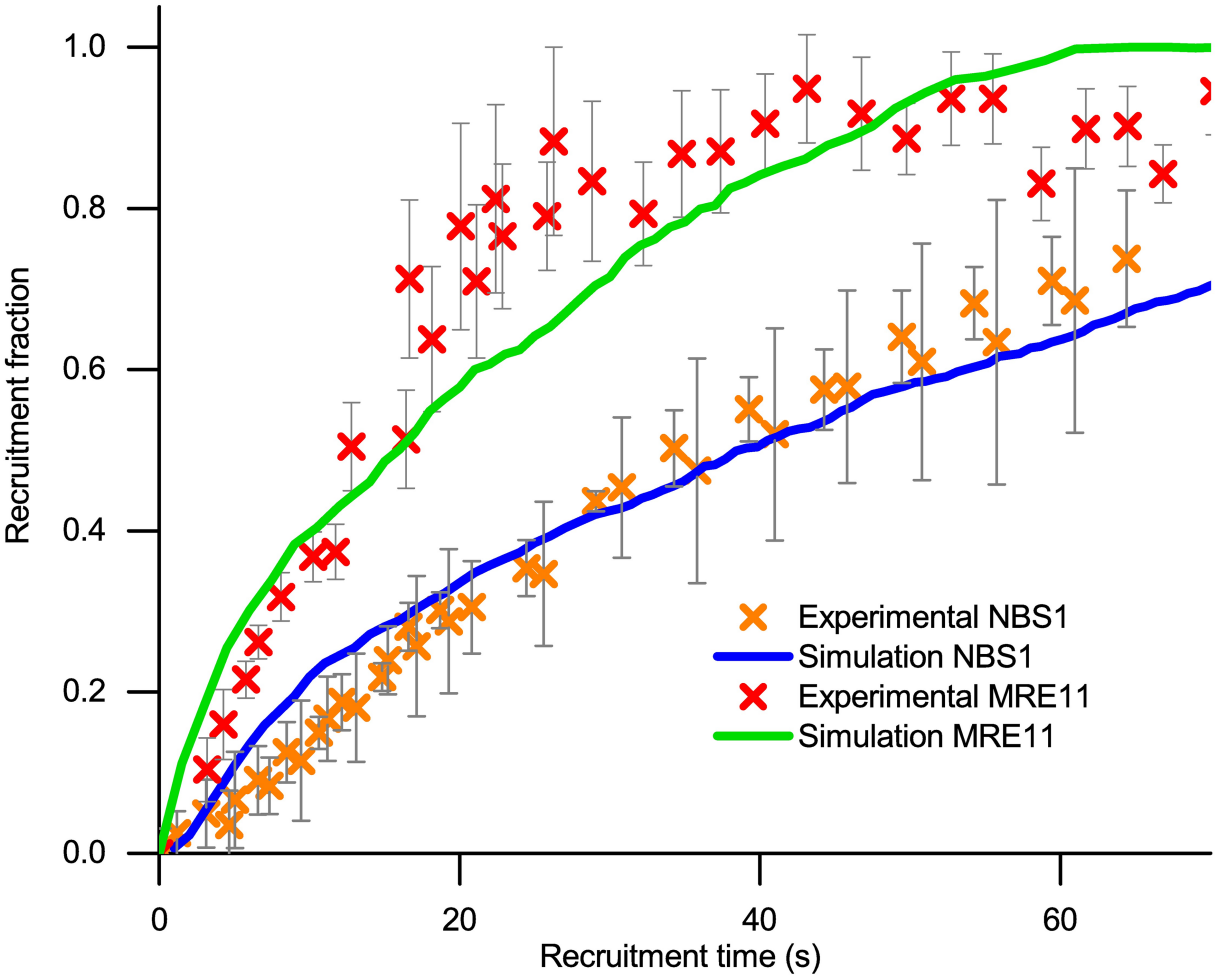
Recruitment of MRE11 and NBS1. The red crosses show the experimental data for recruitment fraction of the MRE11 protein. The orange crosses show the experimental data for NBS1. Both experiments are performed by Haince *et al.* [16]. Numerically, the NBS1 protein has been placed at 14-20 *μ*m from the shell of the nucleus, where the blue line shows the average value of the recruitment time of NBS1. The MRE11 protein has been placed at 11-17 *μ*m away from the nucleus and the green line shows the average value of the recruitment time of MRE11.

The simulation data for NBS1 recruitment and the theoretical distributions agree in the limiting cases describing the diffusion, but the recruitment time is highly dependent on the starting position of the protein, *r*_0_, as illustrated in Fig 4. The figure shows the accumulated fraction of NBS1 in the nucleus, assuming different starting position of the protein. As the time interval for which the theoretical fast diffusion approximation is valid depends on the distance to the target, see Eq (23), the time interval where the asymptotic theoretical solutions that adequately describes the real diffusion becomes shorter, as seen in Fig 4B. The NBS1 protein is produced within a spacial interval in the cell, therefore restricting the simulations to a single starting point would not reflect the actual biological system. To reflect the conditions in an actual cell, an average of different starting points are calculated, assuming a Gaussian distribution with a mean value around 〈*r*_0_〉 = 17 *μ*m. The results are compared with experimental data in Fig 5.

The experimental data [16] is obtained by measuring the accumulation of MRE11 and NBS1 at the DNA damage site, while the numerical equivalent is found as the accumulated number of proteins reaching the nucleus. Theoretically, the accumulation is obtained by integrating the probability density functions to derive Eqs (21) and (19) for fast and slow diffusion regimes, respectively. The experimental results for the fast MRE11 and NBS1 diffusion, plotted in Fig 5, is obtained by Haince *et al.* [16] by measuring the kinetics of MRE11 and NBS1 after a double strand break in mouse embryonic fibroblast and human neuroblastoma. The MRE11 protein was tagged with a yellow fluorescent protein and NBS1 was tagged with green fluorescent protein, allowing recruitment of both proteins to the DNA strand break to be measured as an increase in light intensity.

The derived theoretical expressions cannot describe the actual protein diffusion happening in the cell, first because these formulas were obtained only for the asymptotic assumptions and second due to the restriction regarding a single starting point. To describe the actual recruitment at arbitrary times, simulations are essential.

In this case, the variation of the starting position can be obtained through averaging over the different starting locations of the proteins. The ribosomes are assumed to be normally distributed within the cytoplasm within a region exemplified in Fig 2. Thus, the mean starting position for NBS1 is defined as *r*_0_ = 17 *μ*m, while assuming the possibility of initial position being zero at *r*_0_ = 14 *μ*m and *r*_0_ = 20 *μ*m. For MRE11 the distribution needs to be shifted, at a mean value of *r*_0_ = 14 *μ*m reaching zero at *r*_0_ = 11 *μ*m and *r*_0_ = 17 *μ*m to obtain a reasonable agreement between simulation and experiment results in the recruitment curve that agrees with experimental data for NBS1, as can be seen in Fig 5.

## Discussion

The modeling of DNA double strand break repair is initiated by describing the recruitment of NBS1, MRE11, and RAD50, the proteins responsible for activating the repair mechanism [3, 15]. By setting up a minimal model of the cell whose parameters satisfy the key biological facts, the MRE11 and NBS1 recruitment is modeled as a random walk process and the characteristic time of the process is measured in the limit of fast and slow diffusion. As it is presently unknown if the transport of MRE11 and NBS1 from the ribosome to the nucleus is a diffusive process, the present investigation compares the characteristic MRE11 and NBS1 recruitment time to the theoretical prediction of the first passage time, which is a well-studied property in the field of stochastic processes [29, 36, 37].

The diffusion process is divided into slow diffusion with longer first hitting times and fast diffusion with shorter first hitting times, as the theory can only offer an analytic solution for the asymptotic behaviors. Computer simulations offer an insight in the MRE11 and NBS1 behavior that cannot be described analytically, as the two result only agree when either the short time or long time limits are applied. The numerical results act as expected, as the distribution of NBS1 recruitment times for fast diffusion agrees with the theoretical solution when the time is less than the upper bound determined by Eq (23), and the numerical distribution of recruitment times of the slow solution fits with the theory as well.

The experimental data for MRE11 and NBS1 recruitment agree well with the simulation data, however the starting point of the repair proteins are not consistent, being significantly different. The NBS1 proteins with the higher diffusion coefficient (*D* = 2.5 *μ*m^2^/s) are experimentally found to arrive to the nucleus slower than the MRE11 protein with the lower diffusion coefficient (*D* = 2.0 *μ*m^2^/s). The performed analysis, therefore, suggests that in order to compensate for the slower recruitment, NBS1 would have to be created in ribosomes further away from the nucleus then MRE11. It could be that NBS1 is created in the free ribosomes in the cytoplasm, whereas MRE11 would be created in the ribosomes bound to the endoplasmic reticulum, that is not considered by the simplified theoretical cell model. The endoplasmic reticulum is located in the vicinity of the nucleus, and should MRE11 be created exclusively by the bound ribosomes their initial starting position would be much closer to the nucleus than the distances considered. This hypothesis is worth checking experimentally. Such a difference in synthesis is not unprecedented, as soluble proteins are found to be synthesized in the free ribosomes, whereas membrane proteins are synthesized in the ribosomes bound in the endoplasmatic reticulum [43]. This difference in properties, however, does not seem to be present in the case of MRE11 and NBS1, which are similar proteins. Alternatively, one or both of the transport mechanisms of MRE11 and NBS1 could involve additional factors, e.g. active transport proteins or different penetration properties of the chromatin located within the nucleus, and not only the diffusion originally assumed here.

To further investigate the nature of MRE11 and NBS1 recruitment and by that the time scale of the DNA repair process, a better insight into the exact starting position of each of the repair proteins is needed. Figuring this out would allow for uncovering the method of transportation from the cytoplasm to the DNA breakage site.
